# Seroprevalence and Risk Factors of Inkoo Virus in Northern Sweden

**DOI:** 10.4269/ajtmh.15-0270

**Published:** 2016-05-04

**Authors:** Magnus Evander, Niina Putkuri, Mats Eliasson, Olivia Wesula Lwande, Olli Vapalahti, Clas Ahlm

**Affiliations:** Department of Clinical Microbiology, Virology, Umeå University, Umeå, Sweden; Department of Virology, University of Helsinki, Helsinki, Finland; Department of Public Health and Clinical Medicine, Sunderby Research Unit, Umeå University, Umeå, Sweden; Department of Virology, University of Helsinki, Helsinki, Finland; Helsinki University Hospital, Helsinki, Finland; Department of Clinical Microbiology, Infectious Diseases, Umeå University, Umeå, Sweden

## Abstract

The mosquito-borne Inkoo virus (INKV) is a member of the California serogroup in the family *Bunyaviridae*, genus *Orthobunyavirus*. These viruses are associated with fever and encephalitis, although INKV infections are not usually reported and the incidence is largely unknown. The aim of the study was to determine the prevalence of anti-INKV antibodies and associated risk factors in humans living in northern Sweden. Seroprevalence was investigated using the World Health Organization Monitoring of Trends and Determinants in Cardiovascular Disease study, where a randomly selected population aged between 25 and 74 years (*N* = 1,607) was invited to participate. The presence of anti-INKV IgG antibodies was determined by immunofluorescence assay. Seropositivity for anti-INKV was significantly higher in men (46.9%) than in women (34.8%; *P* < 0.001). In women, but not in men, the prevalence increased somewhat with age (*P* = 0.06). The peak in seropositivity was 45–54 years for men and 55–64 years for women. Living in rural areas was associated with a higher seroprevalence. In conclusion, the prevalence of anti-INKV antibodies was high in northern Sweden and was associated with male sex, older age, and rural living. The age distribution indicates exposure to INKV at a relatively early age. These findings will be important for future epidemiological and clinical investigations of this relatively unknown mosquito-borne virus.

## Introduction

Mosquito-borne viruses cause a variety of clinical syndromes in humans, and they include members of the *Togaviridae*, *Flaviviridae*, and *Bunyaviridae*. The genus *Orthobunyavirus* in the *Bunyaviridae* family has a growing number of strains. Among them, the viruses in the California serogroup are commonly found in the northern hemisphere, particularly in North America, although some―such as Inkoo virus (INKV), Tahyna virus (TAHV), and Snowshoe hare virus (SSHV) or the closely related Chatanga virus―are also present in Europe.^1^–^3^ La Crosse virus of the North American California serogroup is the most well known, causing encephalitis and meningitis in children.^4^ The European TAHV has been associated with an influenza-like illness with signs of meningoencephalitis, although not as severe.

INKV is known to circulate in northern Europe and Russia.^5^–^9^ It has been isolated from *Ochlerotatus communis* mosquitoes in Finland, Sweden, and Russia, and from *Ochlerotatus hexodontus* and *Ochlerotatus punctor* in Russia.^ 10^–^ 12^ Disease associated with INKV infection has not been extensively investigated, but in Russia, the virus has been found to be associated with seasonal fevers (May–September) and infection of the brain (aseptic meningitis, meningoencephalitis, or encephalitis) and even chronic neurological infections.^13^–^15^ Recently, in a study from Finland, acute INKV infection was diagnosed in patients with febrile illness.^9^ The frequency of clinical INKV infection is unknown. However, in Finland, the proportion of the population with anti-INKV IgG antibodies is high: 51% of those tested had anti-INKV IgG antibodies.^9^ Other studies have shown that INKV infection may be present in Alaska but probably not in the European Tyrol Alps.^8^,^16^ Interestingly, the seroprevalence in Finland has increased since the 1960s, when 24% of those tested were seropositive for anti-INKV.^17^

The degree of exposure of the human population to INKV infection in Sweden is not known, so we wanted to investigate the prevalence of anti-INKV IgG antibodies. In this large randomized, stratified, and population-based survey in northern Sweden, we also investigated possible associated risk factors.

## Materials and Methods

### Survey participants.

In this study, we used data from the northern Sweden component of the World Health Organization Monitoring of Trends and Determinants in Cardiovascular Disease (WHO MONICA) study.^18^ Between January and April 2009, a population-based survey was conducted. Subjects aged 25–74 years in the two most northern counties of Sweden (target population 312,000) were randomly selected from population registers and stratified according to age and gender. Thus, for each 10-year age group and starting from the youngest age group of 25–34 years, 250 men and 250 women were invited to participate. Details of sampling and selection have been presented elsewhere.^18^

Of the 2,500 subjects invited, a total of 1,729 participated (69.2%) in the MONICA study in northern Sweden, which was used in the screening for anti-INKV IgG antibodies. Data on individuals who declined participation have been published.^18^ The lowest participation rates were found in the age group 25–34 years: 50.4% in men and 59.6% in women. The corresponding proportions for the age group 35–44 years were 68.4% and 70.8%, for the age group 45–54 years were 67.6% and 70.8%, and for the age group 55–64 years were 76.0% and 75.2%. In the oldest age group, 65–74 years, participation was 78.8% and 74% in men and women, respectively.^18^

Serological samples from 7.4% of subjects were missing. Thus, valid data were available for 1,607 subjects: 50.7% men and 49.3% women.

The measurement procedures included blood pressure measured twice in a sitting position with an interval of 5 minutes. Subjects were weighed on an electronic scale. The highest level of education attained was classified as 1) primary school or secondary school (0–12 years of school) or 2) university studies. Regular smokers smoked at least one cigarette a day; all other subjects were considered to be nonsmokers. Subjects who answered yes to the question, “Do you have diabetes mellitus?” or the question, “Have you been treated for high blood pressure in the past two weeks” in the questionnaire were reported as having “known diabetes” and “hypertension.” Subjects who owned a farm were reported as being “farmers.”

The study was approved by the Regional Ethical Review Board, Umeå, and all subjects gave informed consent.

### Immunofluorescence assay for detection of anti-INKV IgG.

Serum samples obtained from the participants were stored at −80°C before analysis. Anti-INKV IgG antibodies were detected using a previously validated anti-INKV IgG immunofluorescence assay (IFA).^9^ In brief, slides with acetone-fixed Vero E6 cells, 30% of which had been infected with INKV, were incubated with serum at a dilution of 1/20 for 30 minutes at 37°C and then washed with cold phosphate-buffered saline (PBS). The cells were stained with fluorescein isothiocyanate–conjugated antihuman IgG (Jackson ImmunoResearch Laboratories, West Grove, PA) for 30 minutes at 37°C, washed with PBS, and examined with a fluorescence microscope. A positive fluorescence signal from 30% of the cells (with 70% of the cells negative) was required for a positive result.

### Neutralization assay.

A subset of sera (*N* = 90) with negative, weakly positive, or strongly positive INKV IgG results from the IFA were verified with a neutralization assay to determine whether the IgG antibodies were specific to INKV, TAHV, or SSHV.^9^ In brief, serially diluted serum samples were mixed with INKV, TAHV, and SSHV (20 μL of a 500–1,000 plaque forming units/mL suspension) and incubated for 1 hour at 37°C before infection of Vero E6 cells. The neutralization titer was defined as the highest dilution to inhibit the cytopathic effect (CPE). If inconsistency was observed between IFA results and CPE neutralization results, plaque reduction neutralization test (PRNT) was used to confirm the titers by using a reduction in plaque count of 50% as the neutralization endpoint.^3^

### Statistics.

Differences between groups were tested with *t* test or χ^2^ test, and means and proportions were reported. When accounting for differences in sex and age between groups, general linear models were used. All *P* values < 0.05 were considered significant.

## Results

**Figure 1.**
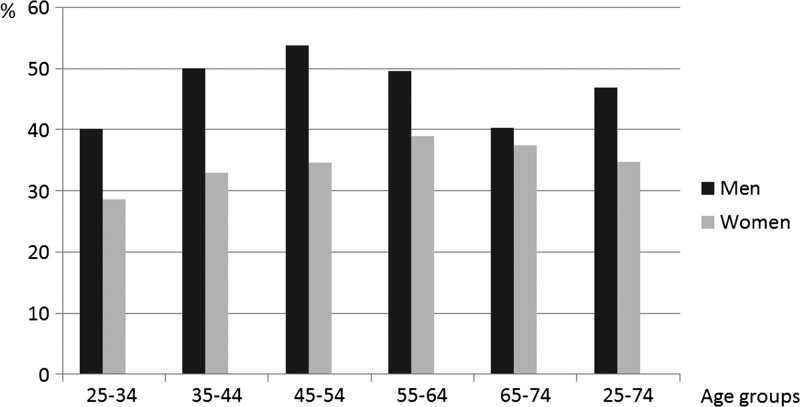
Proportion of anti-INKV IgG antibody positive subjects in Northern Sweden, according to gender and age.

The proportion of subjects who were seropositive for INKV IgG was 40.9%, and it was more common in men (46.9%) than in women (34.8%; *P* < 0.001). The proportion of men who were seropositive varied between the different age groups (*P* = 0.046, χ^2^ test) but there was no linear relationship with increasing age (*P* = 0.6, χ^2^, linear-by-linear association test). In women, the prevalence increased somewhat with age (*P* = 0.06, χ^2^, linear-by-linear) (Figure 1

). There was a peak in seropositivity in the age group 45–54 years in men and in the age group 55–64 years in women (Figure 1). There was no significant difference between the mean age of seropositive subjects and the mean age of seronegative subjects, and this also applied to men and women compared separately.

Living in smaller communities or rural areas was associated with a higher prevalence of seropositivity (*P* < 0.001) (Table 1 ). This pattern was evident in all 10-year age groups, and the observation was statistically significant in ages of 45 years and higher (*P* < 0.03). No other background factors or prevalent disease showed any relationship with previous INKV infection. In an analysis taking gender into account, we found no differences in seropositivity for variables such as level of education, living on a farm, diabetes, and myalgia or arthralgia in either sex with the exception of men living on a small farm (29.4% seropositivity compared with 47.7% in men not living on a farm; *P* = 0.0036). However, the 95% confidence interval for this 18.2% difference in prevalence was 1.2–32%.

Seropositive subjects generally had higher body mass index, waist circumference, systolic and diastolic blood pressure, and serum creatinine (data not shown). After adjusting for the differences in sex and age, no differences between groups remained significant.

Because of the large number of anti-INKV antibody positives, a subset of serum samples (64 positive and 26 negative) was selected for verification of the anti-INKV IgG IFA results by a neutralization assay. The assay involved neutralization of three viruses from the California serogroup: INKV, TAHV, and SSHV. Altogether, 62 of 64 (97%) of the anti-INKV IgG IFA–positive samples, either strongly or weakly positive, specifically neutralized INKV and all anti-INKV IFA–negative serum samples were confirmed to be negative (Supplemental Table 1). As expected, there was some cross-reactivity with TAHV and SSHV. However, anti-INKV titers were approximately 4-fold higher than the antibody titers to the other viruses tested (on average 3.6 and 4.2 higher than for SSHV and TAHV, respectively), with geometrical mean titers of 77 for anti-INKV, 28 for anti-SSHV, and 24 for anti-TAHV). One IFA-positive sample that did not neutralize any of the three viruses was observed, and interestingly, one of the weak IFA-positive samples had a high neutralization titer to TAHV only (Supplemental Table 1). Of the anti-INKV positive samples that neutralized INKV, 11 were further verified by PRNT, and they were all confirmed to be positive (data not shown).

## Discussion

There are numerous kinds of mosquito-borne viruses that may be a threat to public health. Members of the California serogroup are less well known, and there have been very few studies of the epidemiological and clinical properties of the European orthobunyavirus INKV. In this study, we found a high prevalence of anti-INKV antibodies in a population-based cohort from northern Sweden, and more so in men than in women. A similarly high seroprevalence has been shown in a previous study in Finland.^9^

The strength of this study was the use of a large randomized and stratified population-based sample. The only background variable that was significantly associated with seropositivity was living in small communities or rural areas, which suggests more exposure to mosquitoes. All other associations noted could be explained by age and sex differences.

We used a previously validated^9^ serological IFA to detect anti-INKV IgG antibodies. In a subset of samples the results were verified with a neutralization assay, which confirmed the high specificity and sensitivity of the anti-INKV IgG IFA test used for screening.^9^

We cannot completely rule out cross-reactivity to other members of the California serogroup, but positive samples had higher neutralization titers to INKV than to TAHV and SSHV, indicating the presence of anti-INKV antibodies. It is possible that some individuals had undergone infections by other orthobunyaviruses of the California serogroup, either through travel or locally. Interestingly, a SSHV-like Chatanga virus has been identified in the neighboring country Finland.^3^

Intriguingly, the high prevalence of anti-INKV antibodies is not reflected by recognized cases of human infections. The high proportion of seropositive individuals already in the younger age group, 25–35 years, suggests that there is relatively early exposure to this mosquito-borne virus. Interestingly, for the mosquito-borne Sindbis virus, which is also present in northern Sweden, we found that virus antibodies were uncommon in young people, with no age-dependent increase, suggesting a recent introduction of Sindbis virus and increase in this region.^19^ Human INKV infection may be subclinical, and the majority of clinical cases are probably not recognized. In Finland, acute INKV infection has been detected in some patients with suspected viral infections of the central nervous system, but the infection appears to be mild or underdiagnosed.^9^ It is notable, however, that at least one-third of all viral meningitis and encephalitis in Sweden and Finland is of unknown etiology.^20^,^21^

Our results indicate that INKV is widespread and highly endemic in northern Sweden, which may be explained in part by the fact that two of the suggested mosquito vectors, *Ochlerotatus communis* and *Ochlerotatus punctor* are very common^22^,^23^ and that the exposure to mosquitoes, in general, is high in this region.

## Conclusions

In conclusion, the high prevalence of anti-INKV antibodies indicates that this mosquito-borne viral pathogen has been very common in northern Sweden. The seroprevalence was found to be higher in men and in rural areas. These findings draw attention to the possible impact of this infection, which is still relatively unknown to the medical community. However, the clinical importance of INKV requires further studies of febrile cases during the mosquito season in endemic areas. More arbovirus studies are also needed to determine the factors that are important for its circulation and human infection.

## Supplementary Material

Supplemental Table.

## Figures and Tables

**Table 1:** Anti-INKV seropositivity according to demography, lifestyle, and prevalent disease

Variable		Proportion of population with anti-INKV(%)
Size of residential area, no. of inhabitants	> 15,000	35.1
	> 1,000	47.3
	< 1,000	49.2
Level of education	No university studies	41.0
	University studies	40.1
Small farm	No	41.1
	Yes	37.5
Large farm	No	40.9
	Yes	62.5
Daily smoker	Yes	39.4
	No	41.3
Previous myocardial infarction	Yes	40.0
	No	40.9
Previous stroke	Yes	34.9
	No	41.1
Diabetes	Yes	45.5
	No	40.8
Myalgia	No	41.1
	Yes	38.8
Arthralgia	No	40.8
	Yes	42.9
Steroid treatment	Yes	38.3
	No	41.1

INKV = Inkoo virus. All *P* values were > 0.2, except size of residential area (*P* < 0.001).
